# Removing of Disinfection By-Product Precursors from Surface Water by Using Magnetic Graphene Oxide

**DOI:** 10.1371/journal.pone.0143819

**Published:** 2015-12-01

**Authors:** Zhongmou Liu, Xianze Wang, Zhen Luo, Mingxin Huo, Jinghui Wu, Hongliang Huo, Wu Yang

**Affiliations:** 1 School of Environment, Northeast Normal University, Changchun 130117, China; 2 Jilin Engineering Research Centre for Municipal Wastewater Treatment and Water Quality Protection, Changchun 130117, China; 3 Wuxi Xindu Environmental Protection Technology Co., Ltd. Wuxi 214215, China; 4 Scientific Research Management Department, Environmental Monitoring Center of Jilin Province, Changchun 13011, China; Institute for Materials Science, GERMANY

## Abstract

The magnetic graphene oxide (MGO) was successfully synthesised by the in situ chemical co-precipitation method with Fe^3+^, Fe^2+^ and graphene oxide (GO) in laboratory and, was used as an adsorbent for disinfection by-product (DBP) precursors removing from four natural surface water samples. The results indicate that various DBPs formation significantly decreased by 7–19% to 78–98% for the four samples after MGO treatment and, the treatment process was rapidly reached equilibrium within 20 minutes. The DBP precursors removal efficiency decreased with the increasing pH value from 4 to 10. Hydrophobic compounds (humic acid and fulvic acid) are more sensitive to MGO, whereas hydrophilic and nitrogenous compounds (aromatic proteins) are more insensitive. MGO could be regenerated by using 20% (v/v) ethanol and, the DBP precursors removal efficiency can stay stable after five cycles. These results indicate that MGO can be utilized as a promising adsorbent for the removal of DBP precursors from natural surface water.

## Introduction

Disinfection by-products (DBPs) formation in the treatment of drinking water could cause long-term adverse health effects on humans. Trihalomethanes (THMs) and haloacetic acids (HAAs) are the most common DBPs, which are potentially teratogenic, carcinogenic or mutagenic [1].

The formation of DBPs can be controlled and minimised by using one or a combinations of the following approaches: removing of DBP precursors prior to disinfection, changing disinfectants, and removing DBPs after disinfection [2]. Among these methods, the removal of DBP precursors, i.e. natural organic matter (NOM), prior to disinfection is considered the most satisfactory [3]. To date, numerous DBP precursors removal methods have been reported, such as membrane filtration [4,5], activated carbon adsorption [6,7], coagulation [8,9], magnetic ion exchange [10–12], and advanced oxidation processes [13–16]. Among these methods, however, adsorption technology is a high-performance way to control DBP precursors in aqueous solutions due to its low cost, low energy consumption, simplicity of design and high adsorption efficiency.

Graphene oxide (GO), which is an intermediate in the graphene preparation, contains abundant oxygen-containing functional groups on its large surface, such as hydroxyl, carboxyl, and epoxy groups, which makes it extremely hydrophilic and provides it the capability to be used in aqueous environments. GO has been recognized as a superior sorbent for removing environmental contaminants such as tetracycline antibiotics [17], microcystin [18], polycyclic aromatic hydrocarbons [19] and metal ions [20,21]. Though GO exhibits good adsorption properties, it is difficult to separate from aqueous solution, and easy to cause secondary pollution in water for its excellent dispersibility [22,23]. In recent years, magnetic materials have been extensively used in environmental protection due to the fact that magnetic separation technology can separate magnetic materials efficiently and quickly [24,25]. Magnetic graphene oxide (MGO) combines the high adsorption capacity of GO and the easy separation of magnetic particles (Fe_3_O_4_ and γ-Fe_2_O_3_) and could solve the separation problems of GO. MGO has shown beneficial properties for the removal of tetracyclines [26], ionic dyes [27], as well as Cu(II) [28] and Co(II) [29]. However, to our knowledge, there is the absence of information focused on the removal of DBP precursors with MGO.

In this study, MGO was synthesized by chemical co-precipitation and used as an adsorbent to investigate the DBPs control efficiency from four typical surface water samples collected from different water bodies in Changchun, China. Various influence factors of the adsorption process were studied such as dosage, contact time and pH. The regeneration and reusability of MGO were also explored.

## Materials and Methods

### Materials

Flake graphite (99.95%, 325 mesh) was provided by Qingdao Jinrilai Co., Ltd. (Qingdao, China). NH_4_Fe(SO_4_)_2_·12H_2_O and FeCl_2_·4H_2_O were purchased from Sinopharm Chemical Reagent Co., Ltd. (Shanghai, China). Sulfuric acid (H_2_SO_4_, 98%), potassium permanganate (KMnO_4_), hydrogen peroxide (H_2_O_2_) (30%), ammonia solution (25%), hydrochloric acid (HCl) were obtained from Beijing Chemicals Corporation. Sodium hypochlorite (NaOCl, 4%) was purchased from Sigma-Aldrich Chemical Co. (USA). All chemicals used in this study were of analytical grade.

### Water samples

Four typical surface water samples were collected from Ziguang River (ZGR), Yitong River (YTR), Jingyue Reservoir (JYR) and Xinlicheng Reservoir (XLC) in Changchun, China. Samples were filtered through 0.45 μm filter and stored in dark at 4°C for further use. The basic water quality parameters are listed in Table 1.

**Table 1:** Summary of water samples characteristics.

Parameter	YTR	JYR	XLC	ZGR
DOC [mg∙L^-1^]	7.73	5.89	5.56	8.22
UV_254_ [cm^-1^]	0.105	0.073	0.085	0.127
SUVA [L∙mg^-1^∙m^-1^]	1.36	1.24	1.53	1.55
pH	8.1	7.6	7.7	7.4
Br^-^ [μg∙L^-1^]	186	267	207	336

### Preparation and characterization of GO and MGO

GO was synthesized by a pressurized oxidation method described by Bao et al. [30]. The synthesis of MGO was fulfilled by an in situ chemical co-precipitation of Fe^3+^, Fe^2+^ and GO. Firstly, 100 mL of GO (5 mg mL^-1^) were sonicated for 30 min to form stable suspension. Then 8.33 g NH_4_Fe(SO_4_)_2_·12H_2_O and 1.70 g FeCl_2_·4H_2_O were dissolved in 100 mL of ultrapure water under nitrogen protection, followed by rapid addition of 10 mL of 25% ammonia, then the GO suspension was injected drop-wise into the solution with strong stirring and the solution was keep at 85°C for 1 h. The product was collected with a magnet and washed with ethanol and ultrapure water three times, then dried at 65°C for 12 h.

The well prepared GO and MGO were characterized by X-ray diffraction (XRD) (D8 ADVANCE, Bruker, German), fourier transform infrared spectroscopy (FTIR) (Nicolet 6700, Thermo Fisher Scientific, USA), scanning electron microscopy (SEM) (XL30-ESEM, FEI, USA) and transmission electron microscopy (TEM) (TECNAI F20, FEI, USA). Additionally, the zeta potential of MGO was also measured (Nano ZS 90, Malvern, UK).

### Adsorption experiments

0.5 g of MGO were added into 100 ml of the water samples, and shaken at a constant speed at 25 ± 1°C for 100 min. After the adsorption, the adsorbent was collected with a strong magnet, then the water samples were decanted to filter through a 0.45 μm membranes for UV_254_ (T6, Pgeneral, China), DOC (TOC-L CPH, Shimadzu, Japan) and excitation-emission matrix (3DEEM) (F-4500, Hitachi, Japan) analysis. The effect of adsorbent dosages was investigated by adding different amounts of MGO. Moreover, the effect of pH was studied at pH ranging from 4 to 10. All the experimental data were the averages of duplicate determinations, and the relative errors were about 5%.

### MGO regeneration

The separated MGO was firstly washed with 25 mL of 20% (v/v) ethanol for 100 min at 25 ± 1°C, following by washing with 50 mL of ultrapure water twice. This process was repeated 5 times.

### Disinfection by-products formation potential (DBPsFP) analysis

The DBPsFP of the four surface water samples before and after MGO adsorption treatment was analysed. All samples were adjusted to pH 7.0 ± 0.2 with 0.1 M HCl or NaOH. Then, approximately 45 mg∙L^-1^ NaOCl was added and the resulting solutions were incubated in dark at 20°C for 24 h. Sodium thiosulfate was used to neutralize residual chlorine. THMs, haloacetonitriles (HANs) and chloral hydrate (CH) were measured cording to our previous work [31]. HAAs were measured with EPA Method 552.3 by gas chromatograph with an electron capture detector (Clarus 680, PerkinElmer, USA).

## Results and Discussion

### Characterization

S1 Fig in the supplementary data shows the XRD patterns of the GO and MGO. The strongest peaks at 2θ = 11.4° (001) can be assigned to the reflection of the GO, and the peaks at 2θ = 30.3° (220), 35.7° (311), 43.5° (400), 53.9° (422), 57.5° (511) and 63.0° (440) were consistent with the standard XRD data of Fe_3_O_4_. The disappearance of GO diffraction peak (2θ = 11.4°) after the modification with Fe_3_O_4_ can be ascribed to the iron oxides covering up the weak carbon peaks [32].

The FTIR spectra of GO and MGO are shown in supplementary data S2 Fig For GO, O-H groups, C = O groups, C-O-H groups, C-O groups and C = C groups were found in FTIR spectra. Compared with that of GO, the peak of MGO at 561 cm^-1^ was obvious, suggesting that Fe_3_O_4_ nanoparticles loaded to the surface of GO successfully.

**Fig 1:**
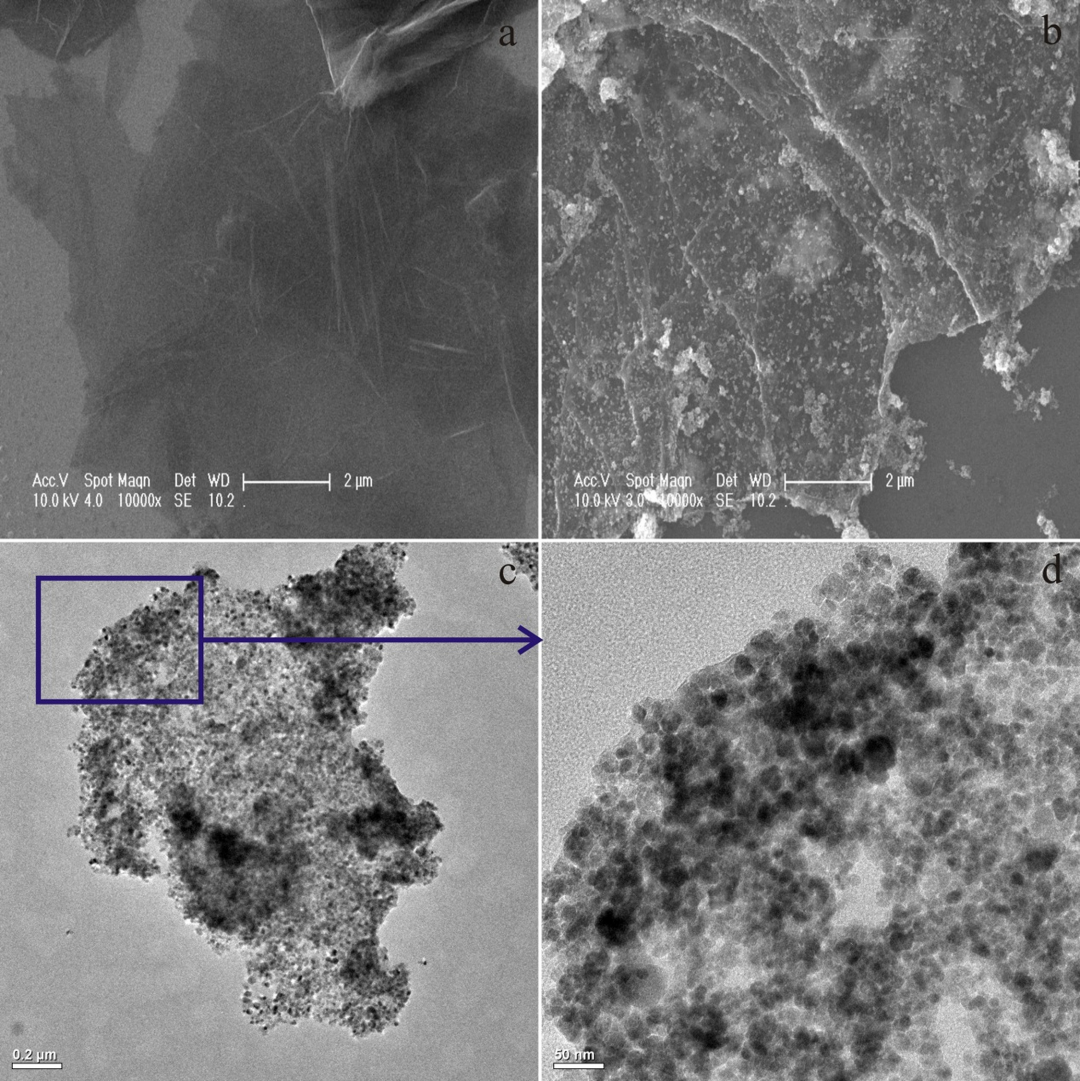
SEM images of GO (a) and MGO (b), TEM images of MGO at low (c) and high resolution (d).

The morphological structure of MGO was observed by SEM and TEM (Fig 1). Compared with the smooth surface and wrinkles of GO (Fig 1A), Fe_3_O_4_ nanoparticles were successfully coated on the surface of GO to form MGO (Fig 1B). Fig 1C and 1D show the TEM images of MGO, in which the results suggested that Fe_3_O_4_ nanoparticles (10–20 nm) were uniformly dispersed on the GO sheets.

Supplementary data S3 Fig shows the zeta potential of MGO at different pH values (4–10), where the isoelectric point (pH_iep_) was approximately 5.4. At pH < 5.4, the surface charge was positive, but was negative at pH > 5.4. When the pH increased, zeta potential of MGO decreased.

### NOM removal studies

Previous works have demonstrated that the abundant oxygen-containing functional groups on the surface of GO can combine with organic matter via hydrogen bonds [33], Lewis acid-base [34], and π-π interactions [35,36].

**Fig 2:**
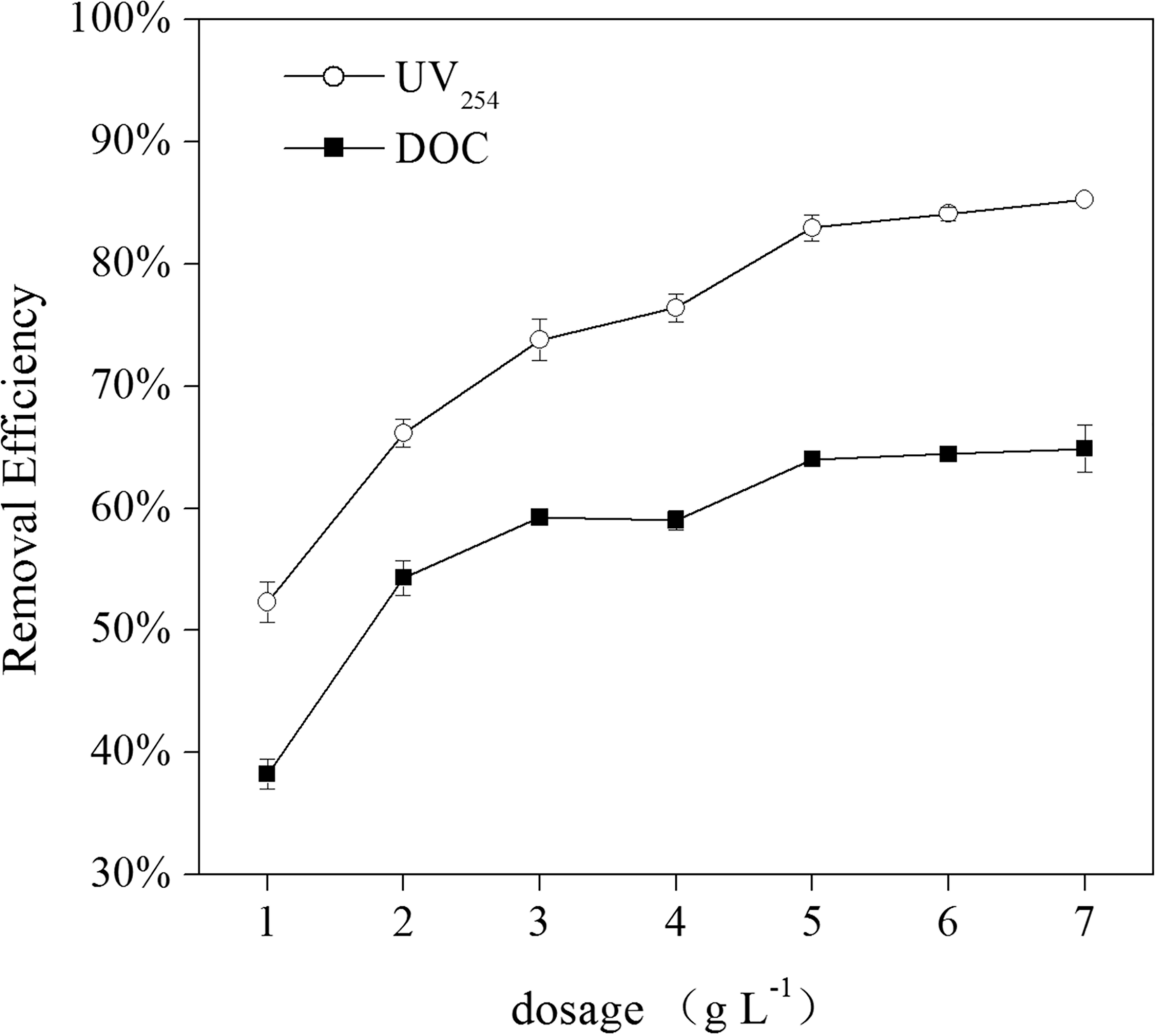
Effect of MGO dosage on the removal efficiency of NOM for ZGR sample (pH = 7.0 ± 0.2, T = 25 ± 1°C).

The ZGR sample was selected as a representative water sample to evaluate the NOM adsorption ability of MGO. Both the UV_254_ and DOC were analysised during the adsorption experiments. As it can be seen from Fig 2, both the UV_254_ and DOC can be efficiently removed by MGO adsorption, and the removal efficiency increased with the increasing MGO dosage. The DOC and UV_254_ removal efficiency increased from 38% and 52% to 64% and 83%, respectively with the increase in the MGO dosage from 1 to 5 g∙L^-1^. This phenomenon illustrated that overall particle surface area increased by adding adsorbent dosage, which resulted in the increase of available adsorption sites, thus increased the adsorption of NOM on the surface of MGO. When the dosge was further increased to 7 g∙L^-1^, no significant increase of the removal efficiency was observed. Thus, 5 g∙L^-1^ of MGO was used in the following experiments.

An interesting result was found that the removal efficiency of UV_254_ was always higher than that of DOC. Wang et al. stated that GO has a significant removal efficiency for hydrophobic aromatic compounds [19]. It was found that hydrophilic organic matter (HiM) was the predominant fraction of DOC, and hydrophobic acid (HoA) fraction was the most important contributor to the UV_254_ [37], thus the removal efficiency of UV_254_ was higher than that of DOC.

**Fig 3:**
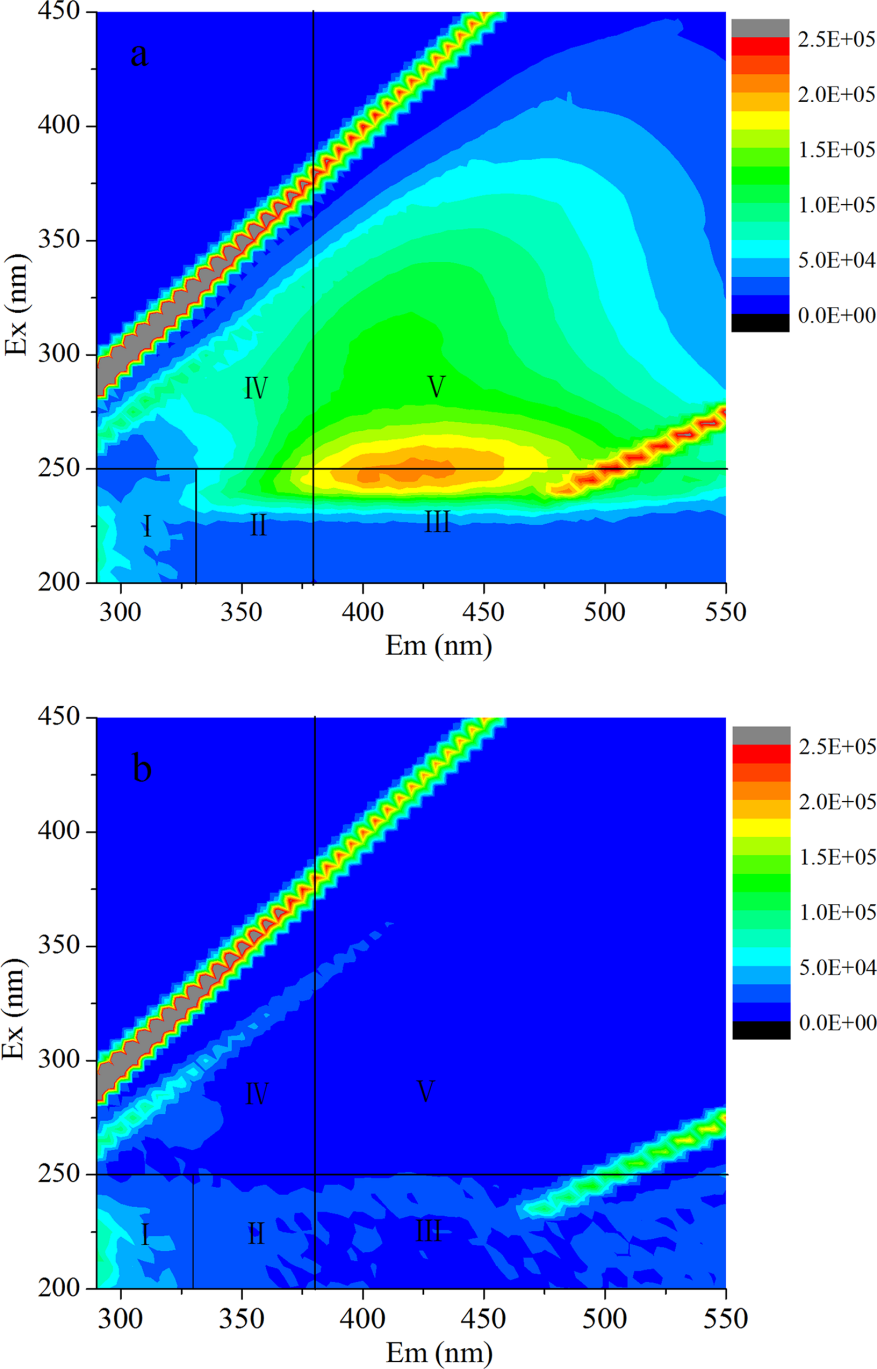
3DEEM fluorescence spectra of ZGR sample before (a) and after (b) MGO adsorption.

The 3DEEM was studied to investigate the NOM removal mechanism. As shown in Fig 3, humic acid (HA), fulvic acid (FA) and soluble microbial byproducts were removed effectively by MGO, the intensity of HA and FA in region III and V were reduced from 2.1×10^5^ to 1.7×10^4^. However, aromatic proteins in region I and II were removed slightly. These findings were consistent with the above result that HoA (HA and FA) was effectively removed by MGO and HiM was difficult to be adsorbed. It suggested that MGO can form a strong π-π stacking interaction with the aromatic rings of the hydrophobic and aromatic organic compounds.

**Fig 4:**
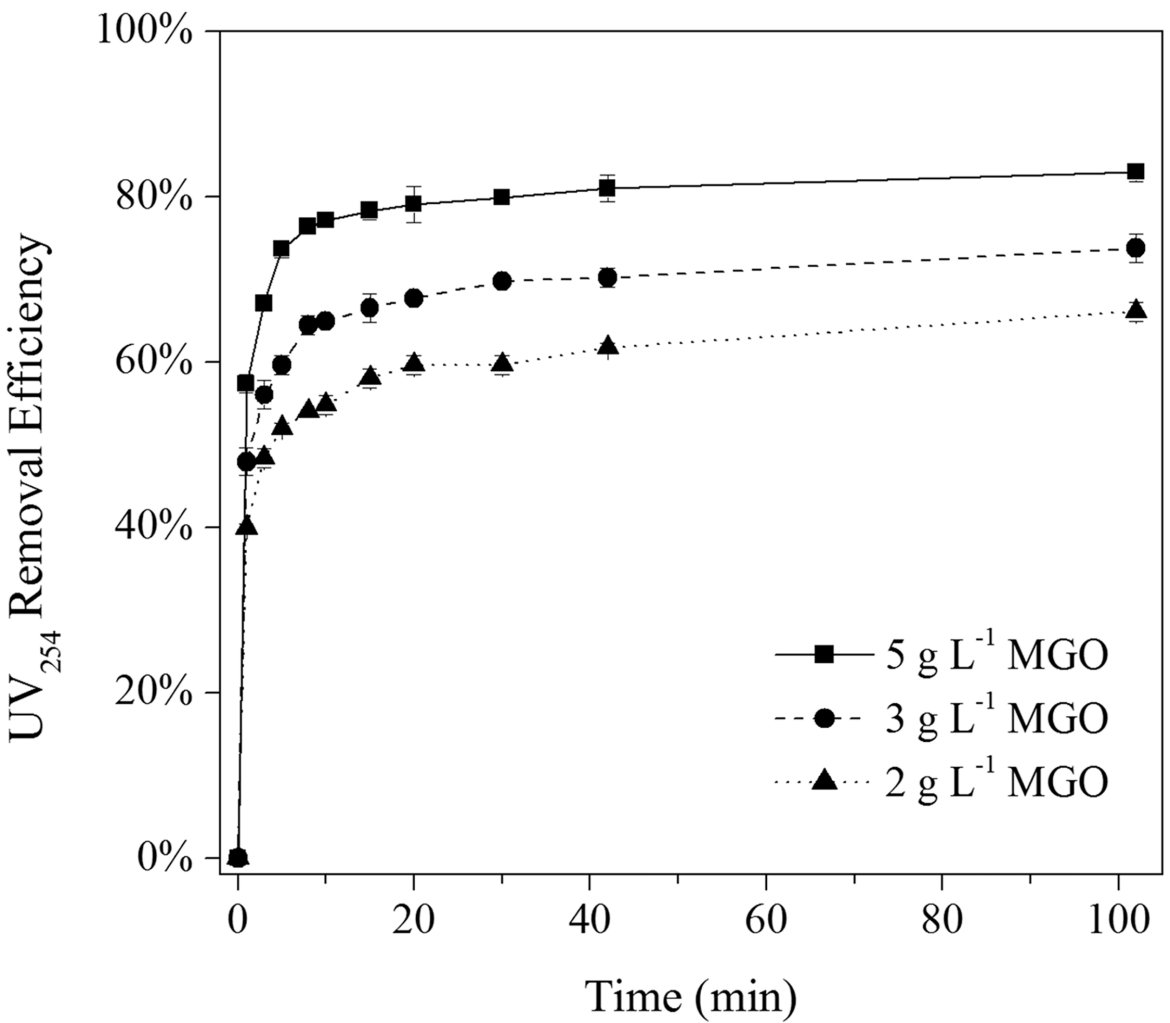
Effect of contact time on NOM removal at different MGO dosages (pH = 7.0 ± 0.2, T = 25 ± 1°C).

Fig 4 shows the effect of contact time on UV_254_ removal at different MGO dosages. It was found that the adsorption process of the NOM mainly occurred in the first 20 min, prolonging the contact time did not significantly increase the NOM removal efficiency. This indicated that MGO had a good adsorption performance, and can reach an adsorption saturation state for organic pollutants. This phenomenon was consistent with previous studies, in which MGO was used to investigate its adsorption properties for organics [38,39].

**Fig 5:**
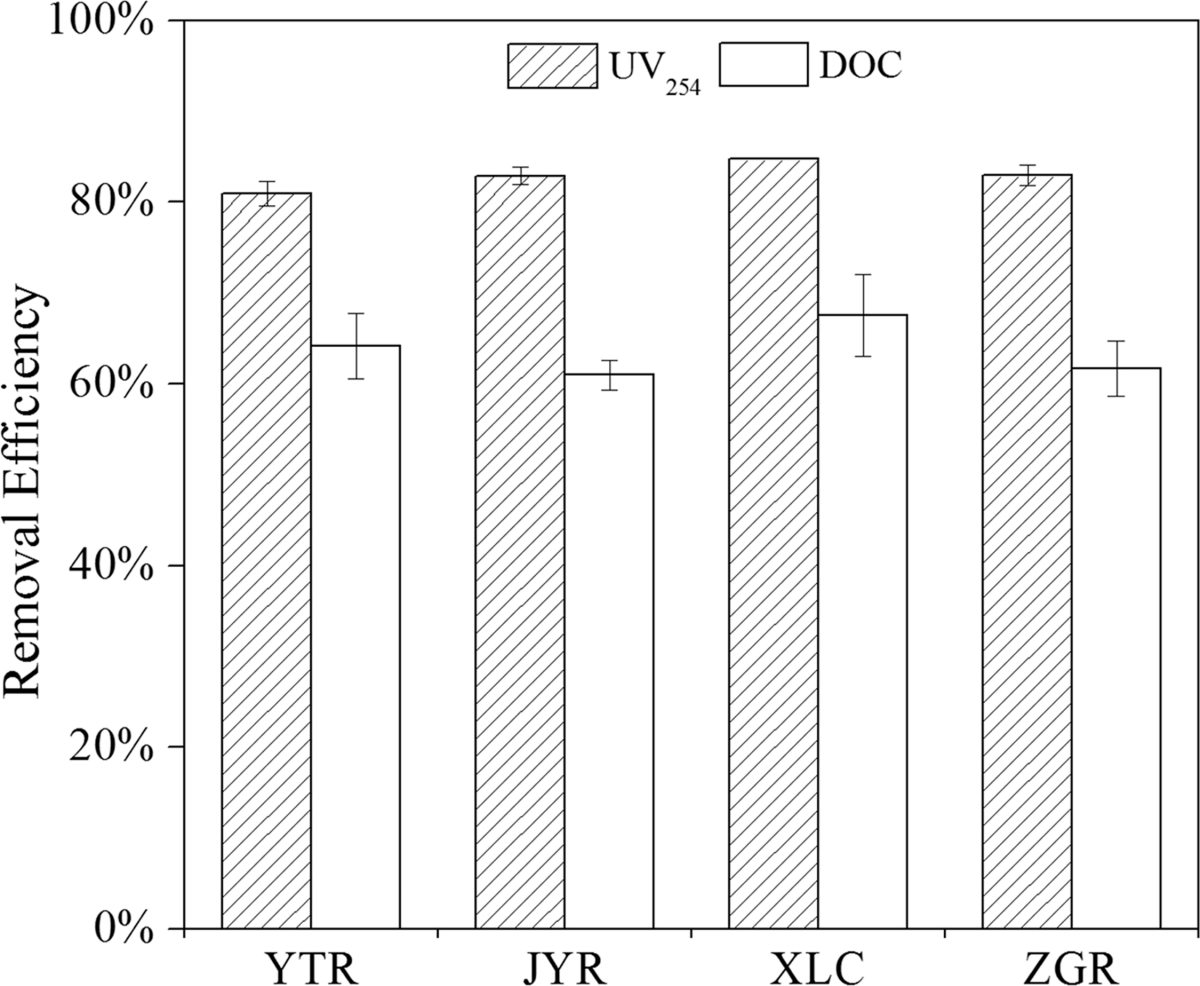
NOM removal efficiency for different surface water samples by MGO adsorption (pH = 7.0 ± 0.2, T = 25 ± 1°C, MGO = 5 g L^-1^).

The rapid processing was important for the application of the MGO composite to remove NOM from natural surface waters in practical application. In order to verify this conclusion, four distinct natural surface water samples were used as sources of DBP precursors for this study. Results for the NOM removal by MGO in the four different surface water samples at a constant pH (Neutral pH) and MGO dosage (5 g L^-1^) are shown in Fig 5. It shows that more than 80% of the UV_254_ and 61–68% of the DOC can be efficiently removed by MGO for all the four samples.

### Effect of pH

**Fig 6:**
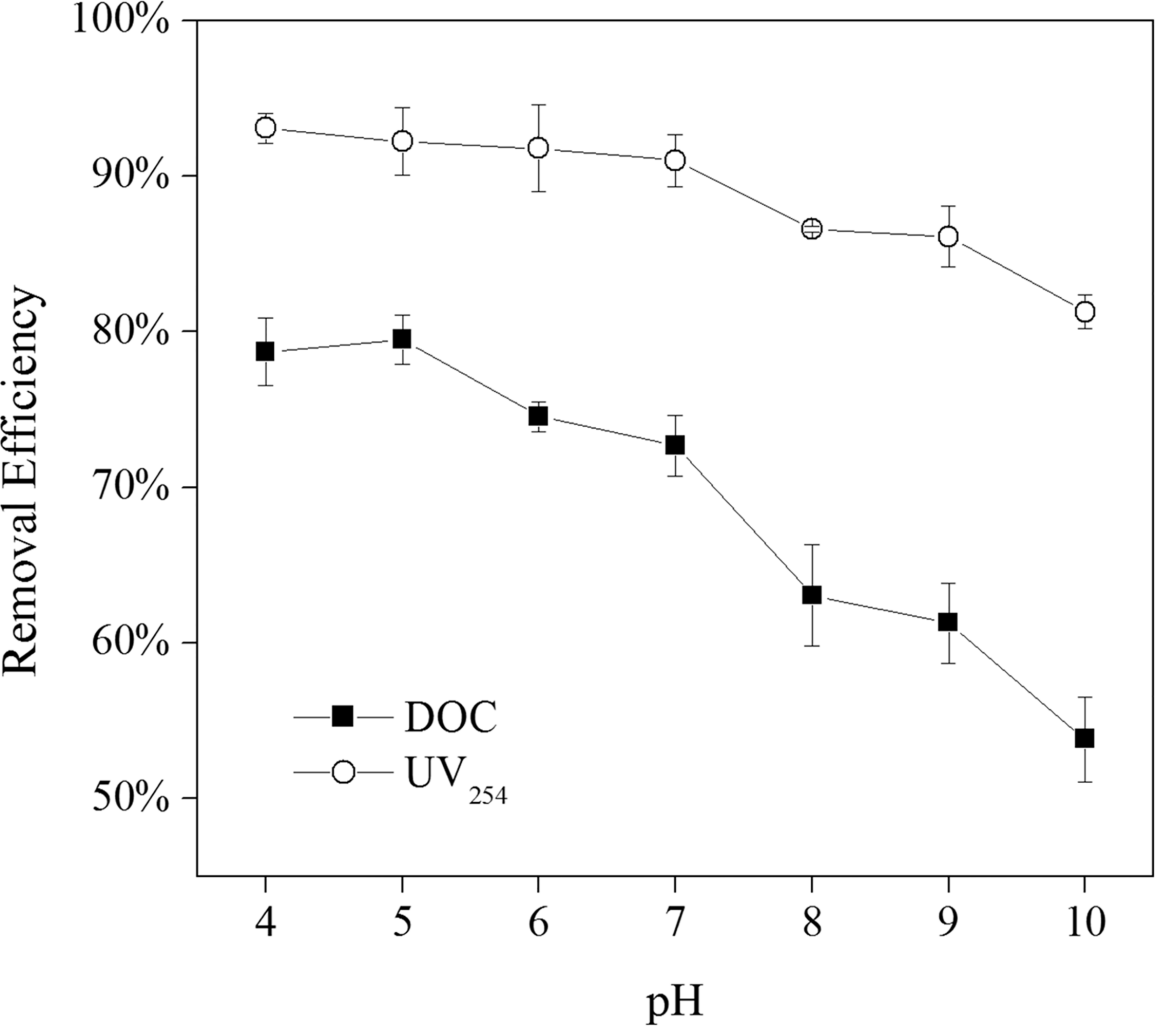
Effect of pH on NOM removal efficiency by MGO adsorption (T = 25 ± 1°C, MGO = 5 g L^-1^).

Given that pH is an essential environmental factor and the most important external factor which can affect adsorption efficiencies, the GO adsorption performances were studied at various pH levels (from 4 to 10). As shown in Fig 6, the removal efficiency of UV_254_ and DOC decreased from 93 to 81% and 79 to 54%, respectively when the pH increased from 4 to 10. In acidic and neutral conditions, the effect of the pH on the removal efficiency was negligible (93 to 91% for UV_254_ and 79 to 73% for DOC). While in alkaline pH ranges, the removal efficiency decreased with the increase in pH. As the pH increased to 10, 81% and 54% of the UV_254_ and DOC was removed. A similar trend has been reported for NOM adsorption using carbon nanotubes [40]. The effect can be attributed by electrostatic interaction mechanism between MGO surface and organics. At different pH, carboxylic and phenolic groups of NOM significantly contributed to the adsorption [41]. In the pH range from 4 to 10, carboxylic and phenolic groups ionized and resulted in a negative charge on the surface of NOM [40–42]. When the pH was lower than the isoelectric point (pH_iep_ = 5.4), the surface charge of MGO was positive due to the protonation reaction effect, thus the electrostatic attraction between the MGO surface and NOM became stronger. When the pH was above 5.4, both the NOM and MGO surface possessed net negative charges, consequently causing an electrostatic repulsion. Though electrostatic repulsion existented, the removal efficiency of NOM should not be ignored, this indicated that the adsorption of NOM onto the surface of MGO may be through other mechanisms. We have previously found that hydrogen bonds, Lewis acid-base, and π-π interactions played a major role.

Due to different pH conditions, Fe_3_O_4_ nanoparticles on the surface of MGO may dissolve releasing ferrum. The concentration of ferrum in treated water was also investigated by inductively coupled plasma spectrometer (ICP) (Prodigy, LEEMAN, USA), and it was found that the dissolved ferrum under the investigated pH ranges was under the detection limit (data not shown), which suggested that MGO possessed good chemical stability and can be safely applied for water treatment.

### Effect of MGO adsorption on DBPsFP

**Fig 7:**
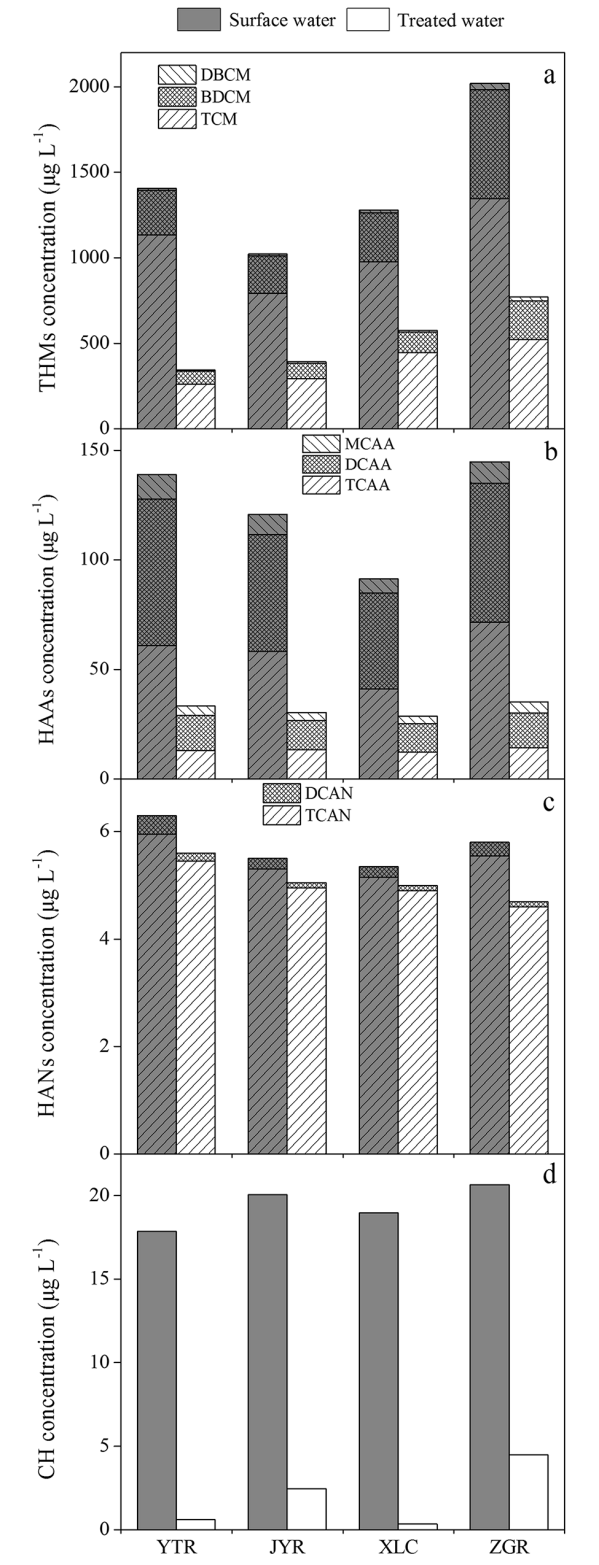
Effect of MGO adsorption on DBPs formation potential of surface water samples (a: THMs, b: HAAs, c: HANs, d: CH).

The DBPsFP before and after MGO adsorption were analysised. As shown in Fig 7, the production of THMs, HAAs, HANs and CH of four water samples after treatment with MGO were reduced 55–76%, 69–76%, 7–19% and 78–98%, respectively. The results demonstrated that MGO is very effective for controlling HAAs, THMs and CH but not for HANs. The following reasons may lead to the above results: (1) MGO can effectively remove hydrophobic carbon, which is the major element in the precursors of THMs and HAAs present in surface water samples [43], (2) MGO may effectively remove aldehydes and hydrophobic amino acids, which are the precursors of CH [44], (3) MGO does not remove a large amount of nitrogenous organic compounds, which are the precursors of HANs. Fig 7A shows that THMs-trichloromethane (TCM), bromodichloromethane (BDCM) and dibromochloromethane (DBCM) can be controlled well. Hua and Reckhow stated that THMs yields had a strong correlation with surface water SUVA values [45]. For the YTR and JYR samples (SUVA < 1.4), the removal efficiency of THMs was: TCM (63–77%) > BDCM (59–71%) > DBCM (28–54%), and for XLC and ZGR (SUVA > 1.5): BDCM (58–64%) > TCM (54–61%) > DBCM (37–43%). The results suggested that the removal efficiency of DBCM did not achieve the desired effect. MGO was effective in removing precursors of TCM at low SUVA values (< 1.4) but not at high SUVA values. The removal efficiencies of the bromine generation precursors were slightly lower than that of the chlorine generation precursors at low SUVA values. Fig 7B shows the removal efficiencies of three kinds of HAAs-trichloroacetic acid (TCAA), dichloroacetic acid (DCAA) and monochloroacetic acid (MCAA) in four samples, which were: TCAA (70%-80%) > DCAA (70%-76%) > MCAA (47%-62%). It can be observed that the precursors of TCAA were mainly composed of HoA and hydrophobic substances (HoS), however, HiM was the major precursor of DCAA and MCAA [37]. This result was consistent with our previous studies that hydrophobic organic compounds were more easily removed by MGO. These results demonstrated the effectiveness of MGO for the removal of DBP precursors, which have great potential application value for the purification of surface water.

**Fig 8:**
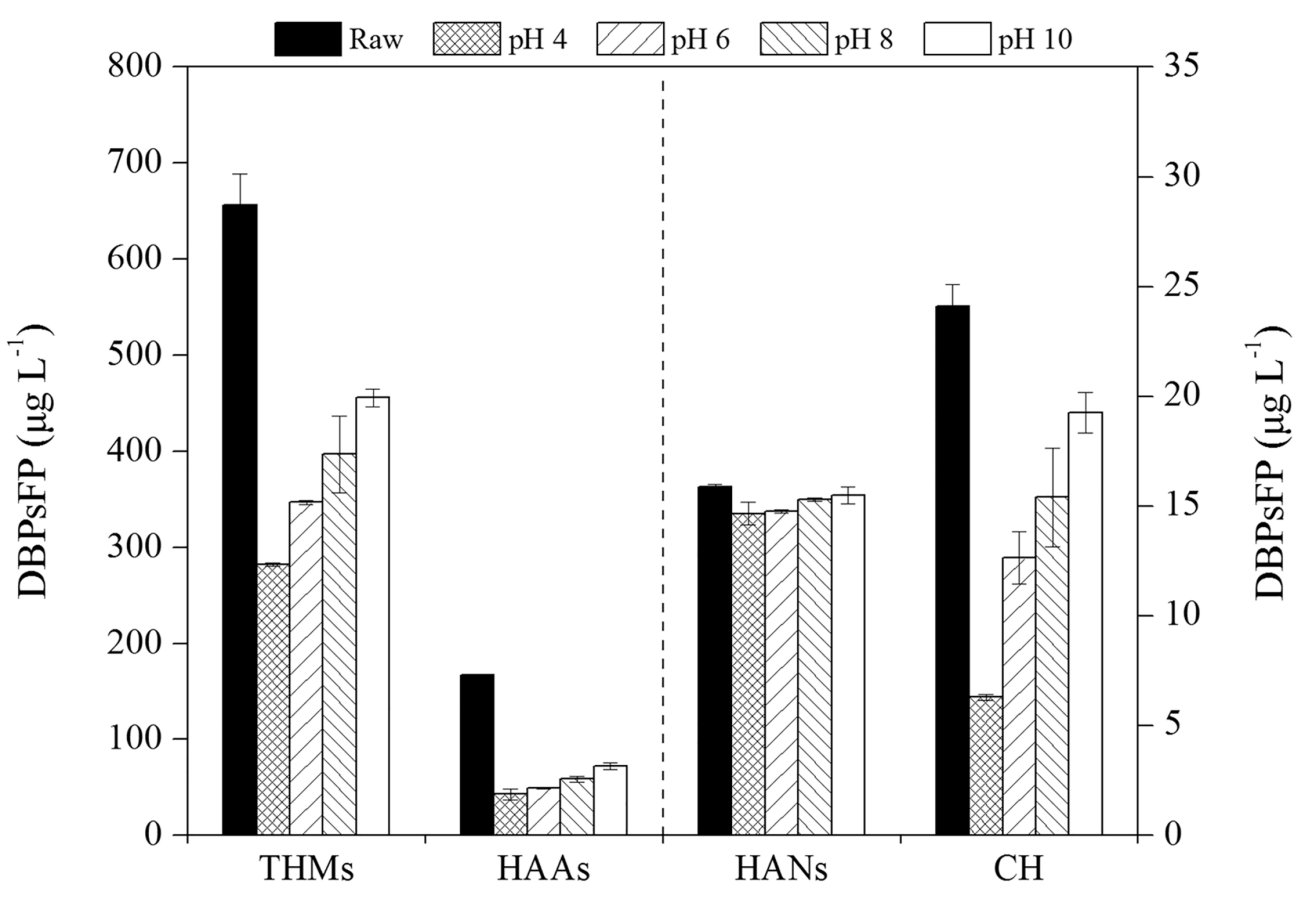
Effect of MGO adsorption on DBPs formation potential of ZGR sample under different pH (pH = 4, 6, 8, 10, T = 25 ± 1°C, MGO = 5 g L^-1^).

The effect of pH on the DBPs formation potential was also investigated by removing precursors in ZGR with MGO. Four DBPs (THMs, HAAs, HANs, CH) formation potential under four pH values (pH 4, 6, 8 and 10) were studied, the results are shown in Fig 8. A deterioration trend of DBPsFP was observed as pH increased. At pH 4, the formation potential of THMs, HAAs, HANs and CH of the treated waters were reduced by 57%, 74%, 8% and 74% respectively. When the pH was increased to 10, the removal efficiency of DBPsFP decreased by 27%, 18%, 5% and 54%, respectively. This result was consistent with the conclusions mentioned in section 3.3, indicating that the DBP precursors adsorption with MGO was strongly dependent on the pH.

### Regeneration and reusability

Ethanol was used for the MGO regeneration due to the fact that it can be used as a cleaning solution in the MGO preparation, and also it can be applied to elute NOM from the surface of MGO and is safe for humans.

**Fig 9:**
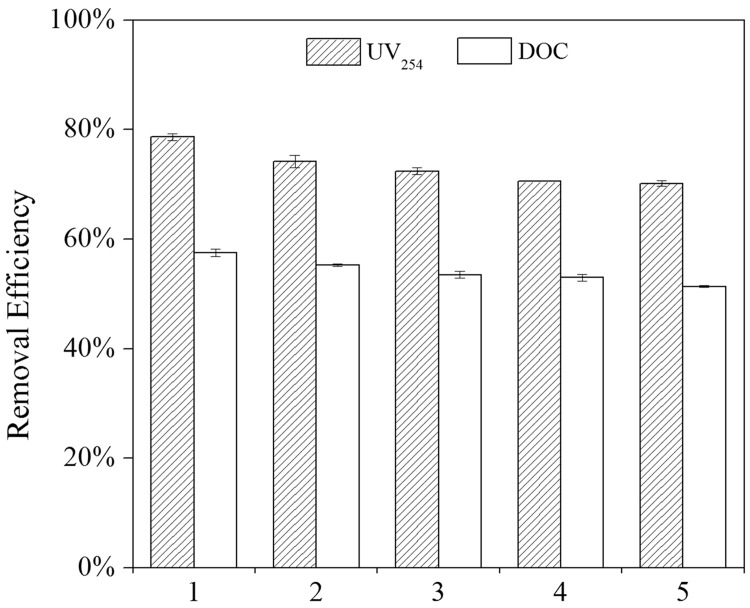
Effect of MGO reuse cycles on NOM removal (pH = 7.0 ± 0.2, T = 25 ± 1°C, MGO = 5 g L^-1^).

In this work, five cycles of MGO regeneration were used for NOM adsorption in ZGR. From Fig 9, it can be observed that the adsorption capacity of NOM decreased when the regeneration cycle numbers increased. After the first regeneration cycle, the removal efficiency of UV_254_ and DOC were 79% and 58%, respectively. After the fifth cycle, the removal efficiency could also reach 70% and 51% for UV_254_ and DOC, respectively. Even though the efficiency decreased, it was not significant. These results demonstrated that MGO could be regenerated effectively by ethanol and have the potential for reusability.

## Conclusions

In summary, MGO was successfully synthetized by the in situ chemical co-precipitation method. MGO can efficiently remove DBP precursors from surface water samples. The removal efficiency of NOM decreased with the increasing pH value from 4 to 10. MGO was suitable for DBP precursors removal from four natural surface water samples and was effective to reduce the formation of THMs, HAAs and CH but not HANs. MGO was effective in the removal of hydrophobic organic compounds in the precursors, but not so effective for nitrogenous organic compounds. MGO can be easily separated by using a powerful magnet and regenerated using 25 mL of 20% (v/v) ethanol. For NOM, the removal efficiencies of UV_254_ and DOC were still able to reach up to 70% and 51% after the fifth washing cycle. It can be concluded that MGO is a very suitable material for controlling DBPsFP in natural surface water.

## Supporting Information

S1 Fig XRD patterns of GO and MGO. (TIF)

S2 Fig FTIR spectras of GO and MGO. (TIF)

S3 Fig Zeta potential of MGO analysed at different pH values. (TIF)

## Abbreviations

MGO: magnetic graphene oxide
GO: graphene oxide
DBP: disinfection by-product
THMs: trihalomethanes
HAAs: haloacetic acids
NOM: natural organic matter
HANs: haloacetonitriles
CH: chloral hydrate
XRD: X-ray diffraction
FTIR: fourier transform infrared spectroscopy
SEM: scanning electron microscopy
TEM: transmission electron microscopy
HiM: hydrophilic organic matter
HoA: hydrophobic acid
TCM: trichloromethane
BDCM: bromodichloromethane
DBCM: dibromochloromethane
TCAA: trichloroacetic acid
DCAA: dichloroacetic acid
MCAA: monochloroacetic acid
HoS: hydrophobic substances
3DEEM: Three-dimensional excitation-emission matrix
