# Supplementary figures and images for: Removing of Disinfection By-Product Precursors from Surface Water by Using Magnetic Graphene Oxide
Source: PLoS One. 2015 Dec 1;10(12):e0143819. doi: 10.1371/journal.pone.0143819 (PMC4666622; doi:10.1371/journal.pone.0143819)

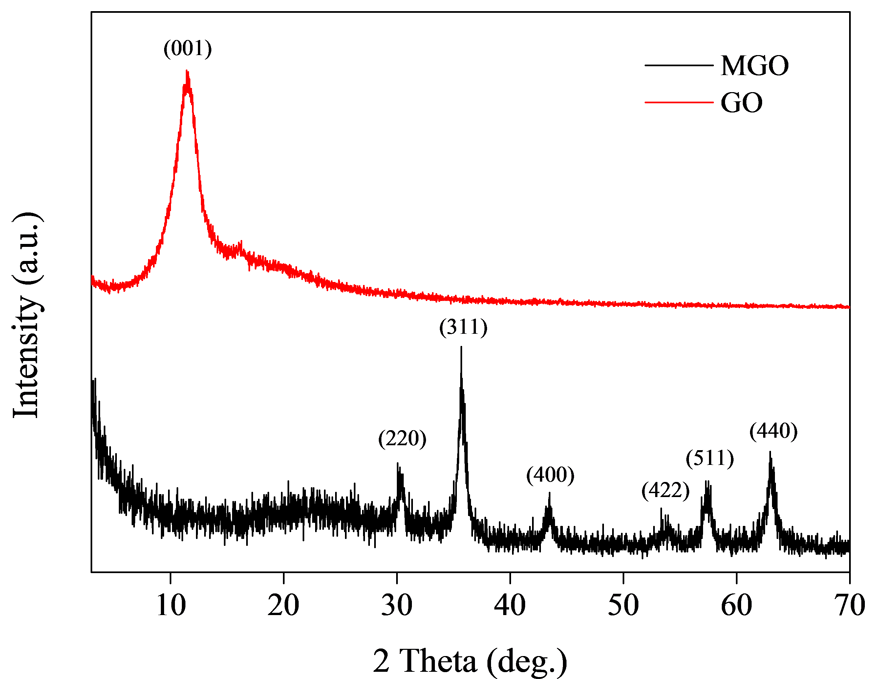

Supplement: S1 Fig — (TIF) [file pone.0143819.s001.tif]

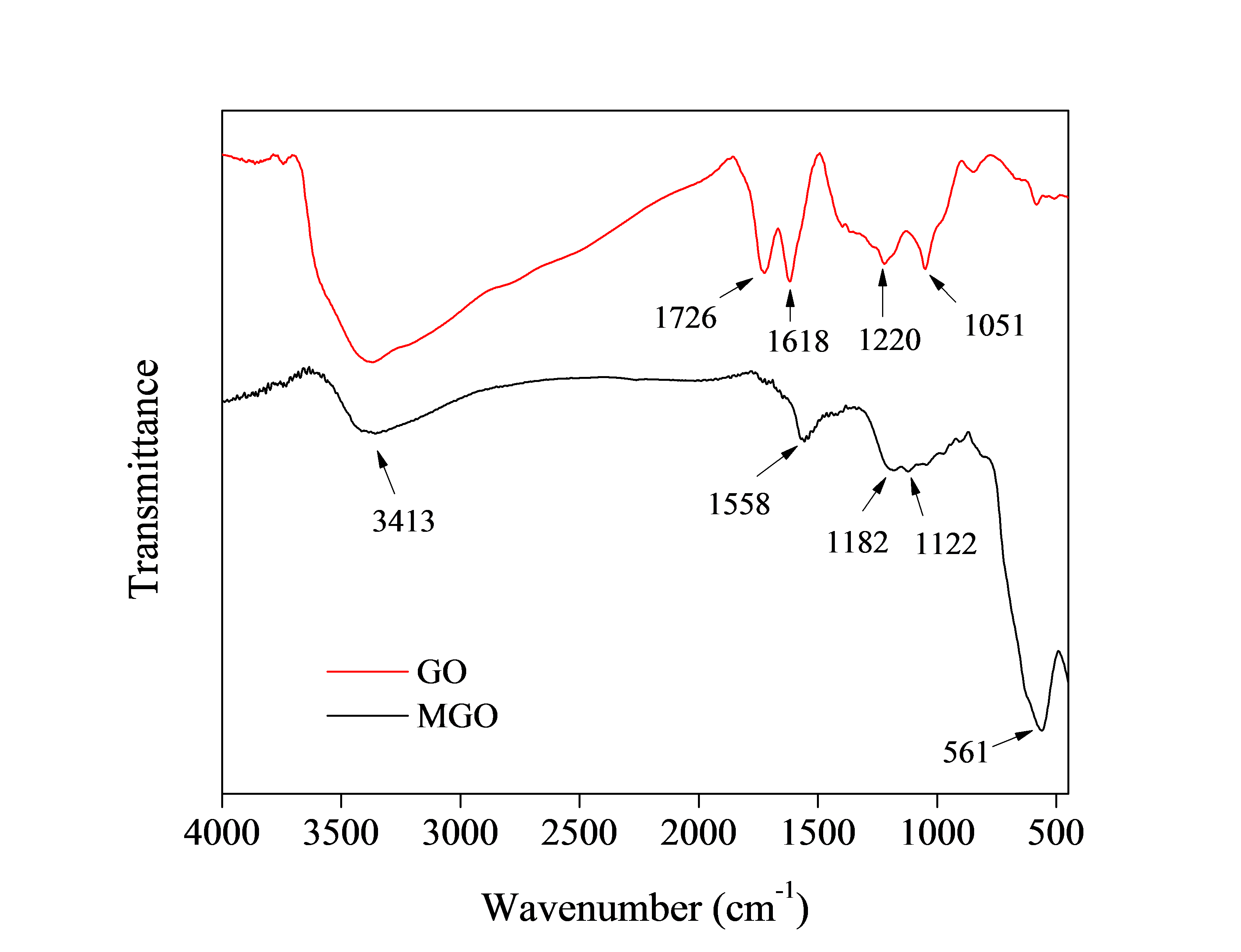

Supplement: S2 Fig — (TIF) [file pone.0143819.s002.tif]

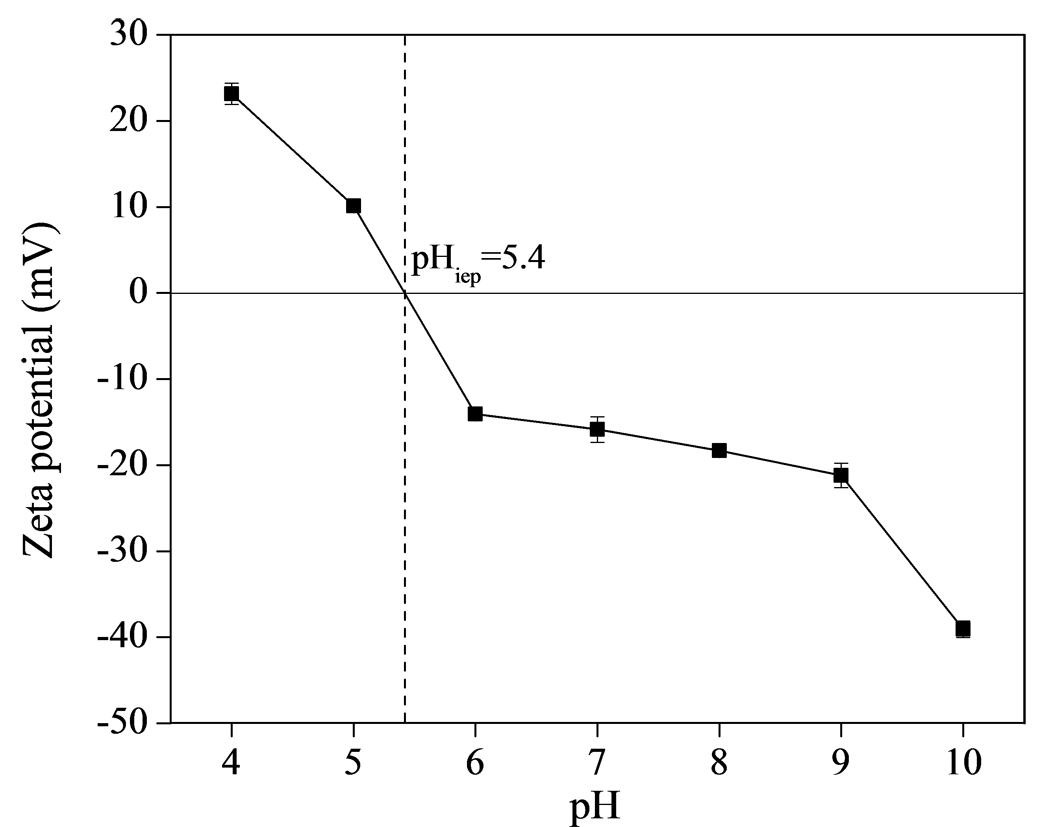

Supplement: S3 Fig — (TIF) [file pone.0143819.s003.tif]
